# Hematologic and biochemical reference intervals for specific pathogen free 6-week-old Hampshire-Yorkshire crossbred pigs

**DOI:** 10.1186/2049-1891-5-5

**Published:** 2014-01-10

**Authors:** Caitlin A Cooper, Luis E Moraes, James D Murray, Sean D Owens

**Affiliations:** 1 Department of Animal Science, University of California, Davis, USA; 2 Department of Population Health and Reproduction, University of California, Davis, USA; 3 Department of Pathology, Microbiology and Immunology, School of Veterinary Medicine, University of California, One Shields Avenue, Davis, CA 95616, USA

**Keywords:** Biochemical analytes, Hematology, Pigs, Reference interval, SPF

## Abstract

**Background:**

Hematologic and biochemical reference intervals depend on many factors, including age. A review of the literature highlights the lack of reference intervals for 6-wk-old specific pathogen free (SPF) Hampshire-Yorkshire crossbred pigs. For translational research, 6-wk-old pigs represent an important animal model for both human juvenile colitis and diabetes mellitus type 2 given the similarities between the porcine and human gastrointestinal maturation process. The aim of this study was to determine reference intervals for hematological and biochemical parameters in healthy 6-wk-old crossbred pigs. Blood samples were collected from 66 clinically healthy Hampshire-Yorkshire pigs. The pigs were 6 wks old, represented both sexes, and were housed in a SPF facility. Automated hematological and biochemical analysis were performed using an ADVIA 120 Hematology System and a Cobas 6000 C501 Clinical Chemistry Analyzer.

**Results:**

Reference intervals were calculated using both parametric and nonparametric methods. The mean, median, minimum, and maximum values were calculated.

**Conclusion:**

As pigs are used more frequently as medical models of human disease, having reference intervals for commonly measured hematological and biochemical parameters in 6-wk-old pigs will be useful. The reference intervals calculated in this study will aid in the diagnosis and monitoring of both naturally occurring and experimentally induced disease. In comparison to published reference intervals for older non SPF pigs, notable differences in leukocyte populations, and in levels of sodium, potassium, glucose, protein, and alkaline phosphatase were observed.

## Introduction

Pigs are emerging as a useful model for studying gastrointestinal (GI) tract and metabolic development and dysfunction [1,2], and may prove to be a particularly good model for investigating the role of GI tract disturbances in inflammatory disease such as inflammatory bowel disease [3] and type 2 diabetes [4]. To better utilize young pigs as a model and understand changes in circulating leukocyte populations and blood chemistry during disease states, reference intervals for clinically healthy, specific pathogen free (SPF), young post-weaning pigs must be established. Reference intervals exist for different ages of pigs including 3-wk-old pigs [5], twelve-wk old pigs [6], and adult pigs [7]. However these pigs are from commercial, non-SPF populations which may be harboring common porcine pathogens [8-10], which could affect the reference intervals. For biomedical research SPF pigs are often used [11,12], and while there are reports of hematological and chemical parameters in SPF mini-pigs [13], there is no reference interval for 6-wk-old SPF pigs that are not derived from miniature pig lines. The aim of this study was to determine reference intervals for hematological and biochemical parameters in healthy 6-wk-old Hampshire-Yorkshire crossbred pigs raised in a SPF facility. The guidelines established by the American Society for Veterinary Clinical Pathology (ASVCP) were utilized to determine the number of animals needed and the correct procedures for determining the reference intervals based on the distribution of each parameter.

## Materials and methods

### Animals

Blood samples were obtained from 66 Hampshire Yorkshire crossbred pigs. The pigs were 6-wks of age and represented both sexes: male (n = 40) and female (n = 26), and weighed between 10–20 kg. All pigs used in this study were examined and considered clinically healthy by a veterinarian. They had normal skin color, body condition and activity.

### Housing

Pigs (*Sus scrofa*) originated from a closed herd and were bred, born, and raised at the University of California swine facility, which is a SPF facility for *Mycoplasma hyopneumoniae*, *Actinobacillus pleuropnemoniae*, porcine reproductive and respiratory syndrome (PRRS) virus, atrophic rhinitis (toxigenic *Pasteurella multocida*), influenza, *Brachyspira hyodysenteriae*, transmittable gastro-enteritis (TGE), *Salmonella typhimurium* and *S. choleraesuis*, internal and external parasites, brucellosis, and pseudorabies virus (PRV). Disease monitoring consists of routine slaughter checks performed by a licensed veterinarian on animals originating from the facility, including lung evaluation and inspection of the nasal passages for signs of atrophic rhinitis. At least four times a yr blood samples collected from adults within the herd undergo serology and PCR analysis at the University of California, Davis, Veterinary Teaching Hospital (VMTH) Clinical Laboratory to screen for all excluded pathogens. Once pigs are weaned, a full necropsy, including screening of feces for pathogens, is conducted on any pig that dies unexpectedly. The necropsies are performed by American College of Veterinary Pathologist (ACVP) board-certified pathologists at the California Animal Health and Food Safety (CAFHS) laboratory (UC Davis, Davis, CA, USA).

### Husbandry

All 66 pigs had their incisor teeth clipped, ears notched, tails docked, and were dosed with 1 mL of oral antibiotic (Spectogard, Bimeda Inc., LeSueur, MN), at 1 d old. At d 3 of age all pigs received an intra-muscular injection of 100 mg iron dextran-200 (Durvet, INC., Blue Springs, MO) and male pigs were castrated. At d 21 of age the pigs were weaned and vaccinated with Fostera (Pfizer Animal Health, New York, NY) for porcine circovirus, then co-housed in mixed litter pens. Once weaned, pigs started to consume Pig A2000 Pellet Denagard/CTC starter diet (Akey, Brookville, OH) containing lactose, cereal food fines, soybean meal, oat groats, ground corn, animal plasma, poultry meal, fishmeal, cheese meal, vegetable and animal fat, and 0.0005% of Lincomix (Pfizer Animal Health, New York, NY) as an antibiotic growth promoter. This diet provided 21% crude protein, 8% crude fat, and 2% crude fiber. Pigs were switched to a standard grower diet (Associated Feed, Turlock, CA) after 2 wk. The grower diet contained wheat millrun, fat mixer, ground corn, blood meal, whole dried whey, soybean meal, Swine Micro 4 mix (Akey, Brookville, OH), and Tylan 40 antibiotic (Elanco Animal Health, Indianapolis, IN) at 0.00004%. This diet provided 20% protein, 7% crude fat, 2% crude fiber, and metabolizable energy of 13.6 MJ/kg. By 6 wks of age pigs weighed between 10 and 20 kg.

### Blood collection

Pigs were placed in a recumbent position on a V shaped table to restrict their movement and blood was collected from the cranial vena cava. Samples for hematologic analysis were collected into 10 mL tubes containing EDTA (Becton Dickinson Company, Franklin Lakes, NJ); samples for biochemical analysis were collected into 5 mL empty serum collection tubes (Becton Dickinson Company, Franklin Lakes, NJ) The use of all animals in this study was approved by the UC Davis Institutional Animal Care and Use Committee, and study subjects were raised under an Association for Assessment and Accreditation of Laboratory Animal Care International (AAALAC) approved animal care program.

### Hematology and blood chemistry

Following collection, blood samples were stored at 4°C before being delivered to the University of California, Davis, Veterinary Teaching Hospital (VMTH) Clinical Laboratory. Samples were analyzed within 4 h of collection. Hematological parameters were analyzed using an ADVIA^®^ 120 Hematology System (Siemens Healthcare Diagnostics Inc., Tarrytown, NY) with a species-specific setting for pigs in the MultiSpecies System Software (Siemens Medical Solutions Diagnostics Inc., Tarrytown, NY, USA). The within-laboratory imprecision for the automated differentials (coefficient of variation, CV) for each variable, as determined by the VMTH Hematology Laboratory, is as follows: RBC 1.0%, HGB 0.8%, MCV 0.4%, RDW 0.6%, WBC 2.7%, and absolute counts for neutrophils 1.6%, lymphocytes 2.9%, monocytes 6.9%, eosinophils 8.8%, basophils 20%, and platelets 2.7%.

Blood chemistry analysis was performed using a Cobas^®^ 6000 C501 Clinical Chemistry Analyzer (Roche Diagnostics, Indianapolis, IN) (Table 1). The CVs for each variable, as determined by the VMTH Clinical Chemistry Laboratory, are as follows: sodium 0.4%, potassium 0.0%, chloride 0.5%, bicarbonate 2.5%, phosphorus 1.3%, calcium 1.1%, BUN 0.7%, creatinine 1.8%, glucose 1.2%, total protein 0.9%, albumin 1.7%, AST 0.8%, creatine kinase 0.8%, alkaline phosphatase 0.5%, GGT 1.0%, total bilirubin 1.7% and SDH-37 2.2%.

**Table 1:** Methods of clinical analysis

**Analyte**	**Method/Principle**	**Reaction type**
Anion gap	Calculated	
Sodium	ISE, diluted	Potentiometry*
Potassium	ISE, diluted	Potentiometry*
Chloride	ISE, diluted	Potentiometry*
Bicarbonate	PEPC/NADH-NAD+	Zero-order kinetic*
Inorganic phosphate	Phosphomolybdate/UV 340 nm	Endpoint*
Calcium	Schwarzenbach/UV 600 nm	Endpoint*
Urea nitrogen	Enzymatic: urease with GLDH	First-order kinetic*
Creatinine	Jaffe	First-order kinetic*
Glucose	Hexokinase	Endpoint*
Total protein	Biuret	Endpoint*
Albumin	Bromocresol green	Endpoint*
Globulin	Calculated	
AST	Modified IFCC	Zero-order kinetic*
Creatine kinase	Modified IFCC	Zero-order kinetic*
Alkaline phosphatase	Modified IFCC	Zero-order kinetic*
GGT	Modified IFCC	Zero-order kinetic*
Bilirubin total	Diazo	Endpoint*
SDH-37	D-Fructose to D-Sorbitol/NADH/UV 340 nm	Zero-order kinetic

All analysis were carried out at 37°C.

AST, aspartate aminotransferase; GGT, γ-glutamyltransferase; SDH-37, sorbitol dehydrogenase; IFCC, International Federation of Clinical Chemistry; ISE, Ion specific electrode.

*Roche Diagnostics GmbH.

### Statistical analysis

The identification of outliers was conducted according to Grubbs [14]. Outliers were removed from the data and all variables were tested for Gaussian distribution using the Shapiro-Wilk test with a significance level of 5%. Variables that were not normally distributed were transformed with square root or log transformations. Reference intervals for such variables were calculated according to the ASVCP guidelines as the sample mean ± 2 standard deviations [15]. For variables that could not be transformed to Gaussian distribution, reference intervals were calculated through a bootstrap procedure. To accomplish this 10,000 bootstrap samples were generated though random sampling and replacement of values in the original dataset. The bootstrap generated standard error and standard deviation were then calculated according to Dimauro et al. [16]. Reference intervals were estimated using the bootstrap mean ± 2 bootstrap standard deviations.

## Results

A total of 66 blood samples for hematologic analysis and 63 blood samples for biochemical analysis were collected. Outliers were identified and removed from the following datasets: hemoglobin, eosinophils, basophils, potassium, glucose, and creatine kinase. Due to a laboratory error the RDW was not measured for 12 samples. The means, medians, minimum and maximum values, reference intervals, and data distributions for the hematological and biochemical parameters are presented in Table 2.

**Table 2:** Hematologic and clinical biochemical reference intervals for 6-week-old Hampshire-Yorkshire pigs between 10–20 kg

**Analyte**	**Unit**	**N**	**Mean**	**Median**	**Min**	**Max**	**Reference interval**	**Data distribution**
**Hematology analytes**								
RBC	m/uL	66	7.31	7.33	5.84	9.04	5.52 - 9.11	Non-Gaussian
HGB	gm/dL	65	10.7	10.8	8.6	12.8	8.8 - 12.7	Gaussian
HTC	%	66	35.5	35.7	25.4	43.8	28.3 - 42.7	Gaussian
MCV	fL	66	48.9	49.8	37.7	59.1	38.4 - 59.3	Gaussian
MCH	pgm	66	14.8	15.3	11.1	18.3	11.1 - 18.4	Non-Gaussian
MCHC	gm/dL	66	30.2	30.3	27.3	32.4	27.9 - 32.4	Gaussian
RDW	%	54	24.4	24.5	16.1	33.3	16.4 - 32.3	Gaussian
WBC	/μL	66	15,315	14,735	5,650	26,470	5,443 - 25,186	Gaussian
Neutrophils	/μL	66	5,668	5,266.5	1,192	15,745	810 - 13,397	Gaussian with square root transformation
Lymphocytes	/μL	66	8,677	8,239	4,045	15,856	3,810 - 14,919	Gaussian with square root transformation
Monocytes	/μL	66	692	619	237	1,460	219 - 1,705	Gaussian with logarithmic transformation
Eosinophils	/μL	64	219	201.5	58	574	45 - 481	Gaussian with square root transformation
Basophils	/μL	64	53	43	11	151	14 - 146	Gaussian with logarithmic transformation
Platelets	/μL	66	540,773	545,500	138,000	909,000	208,588 - 872,957	Gaussian
**Biochemical analytes**								
Anion gap	mmol/L	63	21	20	13	31	14 - 29	Gaussian with logarithmic transformation
Sodium	mmol/L	63	141	140	125	159	131 - 151	Non-Gaussian
Potassium	mmol/L	62	4.9	4.8	3.7	6.3	3.7 - 6.1	Gaussian with square root transformation
Chloride	mmol/L	63	100	100	90	112	93 - 108	Non-Gaussian
Bicarbonate	mmol/L	63	25	25	17	31	19 - 31	Gaussian
Phosphorus	mg/dL	63	8.9	8.8	6.1	12.3	6.3 - 11.5	Gaussian
Calcium	mg/dL	63	11.2	11.2	9.7	12.8	9.9 - 12.5	Gaussian
BUN	mg/dL	63	10	10	4	21	4 - 18	Gaussian with square root transformation
Creatinine	mg/dL	63	0.8	0.8	0.5	1.1	0.5 - 1.1	Non-Gaussian
Glucose	mg/dL	61	106	105	70	139	75 - 136	Gaussian
Total protein	g/dL	63	4.9	4.9	4.1	5.9	4.0 - 5.8	Gaussian
Albumin	g/dL	63	3.9	3.9	3.2	4.7	3.1 - 4.8	Non-Gaussian
Globulin	g/dL	63	1.0	0.9	0.2	1.8	0.3 - 1.7	Gaussian
AST	IU/L	63	44	36	13	141	13 - 111	Gaussian with logarithmic transformation
Creatine kinase	IU/L	62	1,358	921	170	6,823	153 - 5,427	Gaussian with logarithmic transformation
Alkaline phosphatase	IU/L	63	297	280	135	603	130 - 513	Gaussian with square root transformation
GGT	IU/L	63	57	55	34	112	33 - 94	Gaussian with logarithmic transformation
Total bilirubin	mg/dL	63	0.1	0.1	0	0.4	0 - 0.2	Non-Gaussian
SDH-37	IU/L	63	0.5	0	0	4	0 - 1.7	Non-Gaussian

RBC, red blood cells; HGB, hemoglobin; HTC, hematocrit; MCV, mean corpuscular volume; MCHC, mean corpuscular hemoglobin concentration; RDW, erythrocyte distribution width; WBC, white blood cells; BUN, blood urea nitrogen; AST, aspartate aminotransferase; GGT, γ-glutamyltransferase; SDH-37,sorbitol dehydrogenase.

## Discussion

Hematological and biochemical parameters are affected by a variety of factors including age, sex, nutritional and health status, breed, season, and stress [17]. When evaluating results from hematological and biochemical tests these factors must be considered. The 6-wk-old pigs used in this study were all healthy, reared in the same conditions at a SPF facility, fed the same diet, and from a similar genetic background. These genetic, environmental, and nutritional factors should be considered when interpreting the hematological data presented.

The reference intervals of many hematological parameters including HCT, neutrophils, lymphocytes, monocytes, eosinophils, platelets, BUN, glucose, AST, and creatine have a wide range. This high level of variability in the circulating leukocytes is expected because in 6-wk-old pigs those populations are still expanding. The population of pig’s sampled weight ranged from 10 to 20 kg, so parameters such as glucose and creatine which are respectively correlated to adiposity [18] and muscle growth [19], also display a high level of variability.

Pigs are becoming a more common animal model for biomedical research and using SPF pigs helps reduce confounding factors, such as sub-clinical disease, from skewing research results. The results from our study establish reference intervals for both hematological and biochemical parameters in six-wk-old SPF pigs. Six-wk-old pigs are a good animal model because at that age, six-wk old pigs are post-weaning, are undergoing rapid growth, and their immune systems are still maturing. Similarities between porcine and human gastrointestinal and immune system development highlight how the growing pig could represent an important animal model for the study of gastrointestinal and metabolic disease in growing children.

## Competing interests

The authors declare that they have no competing interests.

## Authors’ contributions

CAC: Participated in the design of the study, performed sample collection, and was responsible for drafting the manuscript. LEM: Performed statistical analysis. JDM: Participated in the design of the study. SDO: Conceived of the study, participated in the study design and oversaw sample analysis. All authors read and approved the final manuscript.
